# Serious cardiovascular adverse events with fluoroquinolones versus other antibiotics: A self‐controlled case series analysis

**DOI:** 10.1002/prp2.664

**Published:** 2020-10-12

**Authors:** Sherrie L. Aspinall, Nathan P. Sylvain, Xinhua Zhao, Rongping Zhang, Diane Dong, Kelly Echevarria, Peter A. Glassman, Matthew Bidwell Goetz, Donald R. Miller, Francesca E. Cunningham

**Affiliations:** ^1^ VA Center for Medication Safety Hines IL USA; ^2^ VA Center for Health Equity Research and Promotion VA Pittsburgh Healthcare System Pittsburgh PA USA; ^3^ White River Junction VA Medical Center White River Junction VT USA; ^4^ VA Pharmacy Benefits Management Services Washington DC USA; ^5^ David Geffen School of Medicine at UCLA Los Angeles CA USA; ^6^ VA Greater Los Angeles Healthcare System Los Angeles CA USA; ^7^ VA Center for Healthcare Organization & Implementation Research Bedford MA USA

**Keywords:** adverse drug reactions, fluoroquinolones, Veterans

## Abstract

The objective of this study was to evaluate the association between fluoroquinolone (FQ) use and the occurrence of aortic aneurysm/dissection (AA/AD), acute myocardial infarction (AMI), ventricular arrhythmias (VenA), and all‐cause mortality vs other commonly used antibiotics. We conducted a self‐controlled case series analysis of patients who experienced the outcomes of AA/AD, AMI, and VenA, based on diagnosis codes from emergency department visits and hospitalizations within Veterans Health Administration, and death in FY2014‐FY2018. These Veterans also received outpatient prescriptions for FQs. Conditional Poisson regression models were used to estimate the association between FQs and each of the outcomes vs antibiotics of interest (ie amoxicillin or amoxicillin/clavulanate, azithromycin, doxycycline, cefuroxime or cephalexin, or sulfamethoxazole‐trimethoprim), adjusted for time‐varying covariates. Using a 30‐day risk period after each antibiotic prescription, adjusted incidence rate ratios (aIRRs) for FQs vs each comparator antibiotic were not statistically different for outcomes of VenA or AMI. For AA/AD, incidence was higher during FQ risk periods vs amoxicillin [aIRR 1.50 (95% CI 1.01, 2.25)] and azithromycin [aIRR 2.15 (95% CI 1.27, 3.64)] risk periods. A significantly increased risk of mortality was observed with FQs vs each antibiotic of interest. FQs were associated with an increased risk of AA/AD vs amoxicillin and azithromycin and an increased risk of all‐cause mortality vs multiple antibiotics commonly used for outpatient infections. Although the differences in event rates are small, FQ use should be limited to serious infections without appropriate alternatives.

**Key points**

- In this self‐controlled case series analysis, the incidence of aortic aneurysm/dissection was significantly higher during fluoroquinolone vs amoxicillin and azithromycin risk periods.
- Fluoroquinolones were associated with an increased risk of all‐cause mortality vs multiple antibiotics commonly used for outpatient infections.
- A significantly increased risk of acute myocardial infarction or ventricular arrhythmias was not observed with fluoroquinolones vs each comparator antibiotic.

## 1 INTRODUCTION

Fluoroquinolone (FQ) prescribing has been steadily declining in the Veterans Health Administration (VHA) over the past decade. This is likely due to multiple factors, including widespread antimicrobial stewardship^1^, ^2^ and increased provider awareness of serious adverse drug reactions, such as tendon rupture, irreversible peripheral neuropathy, hypoglycemia, and most recently, aortic rupture and dissection.^3^, ^4^ Although FQ prescribing is decreasing, inappropriate use remains a concern; this includes utilization in patients at increased risk for adverse events.

Following the latest FDA warning in December 2018, the VA Center for Medication Safety, as part of its pharmacovigilance program, performed an active surveillance project to assess the potential association between FQ use and aortic aneurysm/dissection, as well as acute myocardial infarction, ventricular arrhythmias, Achilles tendon rupture, peripheral neuropathy, and 30‐day all‐cause mortality in Veterans. Using propensity score matching with readily available potential confounders and Cox Proportional Hazards regression, the surveillance project detected a potential signal of an increased risk of aortic aneurysm/dissection, acute myocardial infarction, and 30‐day all‐cause mortality with FQs compared to both azithromycin and amoxicillin. Therefore, a more comprehensive and rigorous study was conducted using methods that would adjust for additional potential confounding. The objective of this study was to evaluate the association between FQs and the occurrence of aortic aneurysm/dissection (AA/AD), acute myocardial infarction (AMI), ventricular arrhythmias (VenA), and all‐cause mortality vs other commonly used antibiotics employing a self‐controlled case series analysis (SCCSA).

## 2 MATERIALS AND METHODS

### 2.1 Study design and sample construction

Self‐controlled case series analysis is useful when the exposure is transient and the outcome acute.^5^, ^6^, ^7^ Patients serve as their own controls, so only cases are included, and there is no need to adjust for time‐invariant or fixed confounders (eg sex, race/ethnicity). The analytic method allows for adjustment of time‐varying covariates and can include multiple exposures. However, patients must have both the outcome and the exposure of interest. Our design is very similar to that employed by DiDiodato and Fruchter in an SCCSA of antibiotic exposure and the risk of *Clostridium difficile* infection.^7^

**FIGURE 1:**
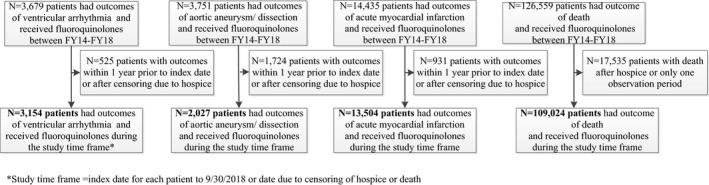
Sample construction

Veterans aged ≥18 years who had the outcomes of VenA, AA/AD, and AMI based on International Classification of Diseases, Ninth and Tenth Revisions, Clinical Modification (ICD‐9/10‐CM) diagnosis codes (Table S1)^8^, ^9^, ^10^, ^11^, ^12^ in the primary or principal position for emergency department visits or hospitalizations, respectively, or death during the study period of fiscal years (FYs) 2014 through 2018 *and* received oral FQs as outpatients in this same time frame were eligible for inclusion (Figure 1). Patients who received >42 consecutive days of fluoroquinolones (ie chronic therapy) were excluded. The study time frame started one year after their first inpatient stay or outpatient visit to ensure a full baseline year; this was the index date (earliest date was 10/1/2013). The evaluation ended on the date the patient entered hospice/palliative care, the date of death, or the end of the study period (ie 9/30/2018), whichever was earliest. The Institutional Review Boards for VA Pharmacy Benefits Management Services and VA Pittsburgh Healthcare System approved the study.

### 2.2 Outcomes

Patients could enter multiple outcome groups and have the same outcome more than once, except for death. Patients with any of the specified ICD‐9/10‐CM diagnosis codes, in any position, associated with an inpatient or outpatient visit within one year prior to the index date were excluded to try to identify incident events. For patients with the outcome of death, those with only one observation period (ie periods of time when patient is at risk of an outcome due to receipt of an antibiotic or periods of time when patient is not at risk because no antibiotics were received) were excluded because SCCSA (within person comparisons) could not be conducted (Figure 1).

### 2.3 Data sources

Data on demographics, comorbidities, inpatient/outpatient encounters for VenA, AA/AD, and AMI, and common respiratory, urinary, and skin and soft‐tissue infections were obtained from the Inpatient and Outpatient Medical SAS datasets in the National Patient Care Database. The Pharmacy Benefits Management (PBM) Services outpatient prescription database (v 3.0) was used to extract data on outpatient antibiotic prescriptions; smoking status was coded using Corporate Data Warehouse Health Factors data, and the Vital Status file was used to identify date of death.

### 2.4 Comparator antibiotics

We compared risks of VenA, AA/AD, AMI, and death in patients who received oral FQs (ie levofloxacin, ciprofloxacin, moxifloxacin) vs five antibiotic groups, amoxicillin or amoxicillin‐clavulanate (ie amoxicillin group), azithromycin, doxycycline, cefuroxime/cephalexin, and sulfamethoxazole‐trimethoprim.

### 2.5 Construction of observation periods

**FIGURE 2:**
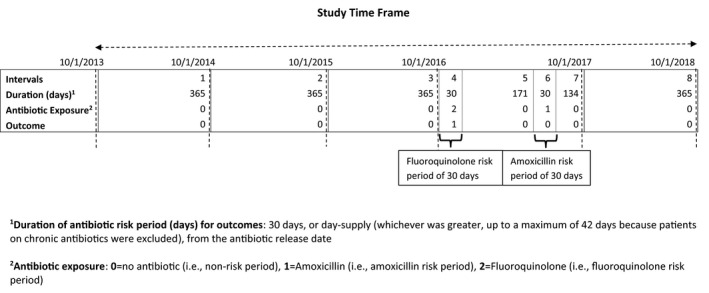
Example antibiotic risk and non‐risk periods for outcomes

Observation periods included risk periods and non‐risk periods (ie no antibiotic). The antibiotic risk periods were 30 days,^8^, ^13^ or entire day‐supply (whichever was greater, up to a maximum of 42 days), from the antibiotic release date (Figure 2). If a second prescription for the same antibiotic was released within the risk period of the first antibiotic (eg day 7), the risk period continued for 30 days from the release date of the second prescription. However, the risk period for the first antibiotic ended when a second prescription for a different antibiotic was released within the risk period of the first prescription. The observation periods were classified into nine categories and accounted for all person‐study time, including risk periods for FQs, each of the five comparator antibiotic groups of interest, other individual antibiotics (ie antibiotics not of interest such as nitrofurantoin), and multiple antibiotics (ie overlapping risk periods of ≥2 antibiotics), as well as a non‐risk period (ie no antibiotics).

### 2.6 Time‐varying and fixed covariates

To describe the patients who experienced each of the outcomes, we collected data on demographics (ie age, sex, race/ethnicity), smoking status, and comorbidities, as defined in the Quan coding algorithm for the Charlson Comorbidity Index (CCI), at baseline.^14^ We also pulled data on several comorbidities that are risk factors for the outcomes of interest, but not in the CCI. Our time‐varying covariates included age, fiscal year of index date, and common respiratory (ie pneumonia, chronic obstructive pulmonary disease exacerbation, bronchitis, pharyngitis, sinusitis, cough, upper respiratory infection), urinary (ie urinary tract infection, pyelonephritis, prostatitis, bacteriuria), and skin and soft‐tissue (ie cellulitis, skin abscess, diabetic foot infection, skin and soft tissue) infections associated with receipt of outpatient antibiotics. These infections were identified using ICD‐9/10‐CM diagnosis codes, in any position, associated with outpatient or emergency department visits or hospitalizations within seven days before the start of the antibiotic risk period through seven days after (Table S1).

### 2.7 Statistical analysis

We described patient characteristics for each of the four outcome analysis samples and summarized the proportion of patients on each antibiotic by the four outcomes. For each risk and non‐risk period, we summarized the total number of events, the total number of person‐days, and the event rate per 100 person‐days. Conditional Poisson regression models were used to estimate the association between FQs and each of the outcomes vs the antibiotics of interest using within person comparisons, adjusted for the time‐varying covariates. Results are presented as adjusted incidence rate ratios (aIRRs) and 95% confidence intervals. The IRR is a ratio of the incidence rate of the outcome in the FQ risk period compared with the incidence rate of the outcome in another antibiotic risk period (eg amoxicillin, azithromycin). For completeness, aIRRs for FQs vs no antibiotics, other individual antibiotics, and multiple antibiotics are presented. In addition, we calculated aIRRs for FQs vs all antibiotics combined (ie antibiotics of interest and other antibiotics) for each outcome as a comparison with the primary results. A sensitivity analysis was conducted by running the Poisson regression models after removing patients who had >1 outcome of the same type. We also examined a 10‐day risk period for all outcomes and 60 days for AA/AD as sensitivity analyses.^9^, ^11^, ^12^ A two‐sided *P* < .05 was used to indicate statistical significance. Analyses were performed using SAS version 9.4 (SAS Institute Inc, Cary, NC) and STATA 14 (College Station, TX).

## 3 RESULTS

The outcome groups included 3154 patients with VenA, 2027 patients with AA/AD, 13 504 patients with AMI, and 109 024 patients who died *and* received at least one outpatient prescription for a FQ (Figure 1). Table 1 includes the proportions of patients who received prescriptions for each of the antibiotics, by outcome. For all four outcome groups, patients were predominantly male, and the majority were white (Table 2). At baseline, the mean age of patients was approximately 68 years old for all outcomes, except mortality, which was 72.5 years. The percentage of current smokers was 36%‐38% in all outcome groups, except AA/AD, in which it was 53%.

**TABLE 1:** Proportion of patients, by outcome, who received outpatient prescriptions for the comparator antibiotics during the study time frame

	Ventricular arrhythmia N = 3154 n (%)	Aortic aneurysm/dissection N = 2027 n (%)	Acute myocardial infarction N = 13 504 n (%)	Mortality N = 109 024 n (%)
Fluoroquinolone	3154 (100)	2027 (100)	13 504 (100)	109 024 (100)
Amoxicillin	1539 (48.8)	809 (39.9)	6232 (46.1)	33 475 (30.7)
Azithromycin	1075 (34.1)	571 (28.2)	4556 (33.7)	22 627 (20.8)
Cefuroxime/Cephalexin	1142 (36.2)	570 (28.1)	4399 (32.6)	22 757 (20.9)
Doxycycline	1097 (34.8)	496 (24.5)	4109 (30.4)	19 325 (17.7)
SMX‐TMP	929 (29.5)	540 (26.6)	3655 (27.1)	22 552 (20.7)
Other oral antibiotics	1469 (46.6)	759 (37.4)	5968 (44.2)	32 818 (30.1)

Note

SMX‐TMP, sulfamethoxazole‐trimethoprim.

**TABLE 2:** Baseline patient characteristics by outcome

	Ventricular arrhythmia	Aortic aneurysm/dissection	Acute myocardial infarction	Mortality
Patient characteristics	N = 3154 patients n (%)	N = 2027 patients n (%)	N = 13 504 patients n (%)	N = 109 024 patients N (%)
Male	3022 (95.8)	1992 (98.3)	13 088 (96.9)	105 886 (97.1)
Race/ethnicity				
Hispanic	245 (7.8)	138 (6.8)	1439 (10.7)	8510 (7.8)
White	1815 (57.5)	1390 (68.6)	7952 (58.9)	72 768 (66.7)
Black	969 (30.7)	416 (20.5)	3536 (26.2)	23 289 (21.4)
Asian	48 (1.5)	43 (2.1)	287 (2.1)	1936 (1.8)
American Indian/Alaska Native	51 (1.6)	31 (1.5)	205 (1.5)	1331 (1.2)
Unknown	26 (0.8)	9 (0.4)	85 ( 0.6)	1190 (1.1)
Age (mean, sd)	66.6 (10.7)	68.6 (8.8)	68.4 (10.7)	72.5 (11.4)
18‐39	59 (1.9)	9 (0.4)	69 (0.5)	622 (0.6)
40‐64	1276 (40.5)	686 (33.8)	5046 (37.4)	28 265 (25.9)
65‐84	1666 (52.8)	1234 (60.9)	7297 (54.0)	62 025 (56.9)
85+	153 (4.9)	98 (4.8)	1092 (8.1)	18 112 (16.6)
Smoking status^a^				
Current	1203 (38.1)	1087 (53.6)	4856 (36.0)	39 507 (36.2)
Former	765 (24.3)	425 (21.0)	3622 (26.8)	28 046 (25.7)
Never	484 (15.3)	165 (8.1)	2150 (15.9)	15 237 (14.0)
Unknown	702 (22.3)	350 (17.3)	2876 (21.3)	26 234 (24.1)
Charlson Comorbidity Index^b^ (mean, sd)	2.2 (2.1)	1.4 (1.7)	2.3 (2.2)	2.5 (2.3)
Other comorbidities^b^				
Cardiomyopathy	339 (10.7)	55 (2.7)	583 (4.3)	4288 (3.9)
Hypertension	2336 (74.1)	1376 (67.9)	10 559 (78.2)	78 936 (72.4)
Atherosclerosis	87 (2.8)	40 (2.0)	576 (4.3)	3418 (3.1)

^a^ Smoking status within 2 y prior to baseline

^b^ Comorbidities within 1 y prior to baseline

Using a 30‐day risk period, the aIRRs for FQs vs each comparator antibiotic of interest for the outcomes of VenA and AMI were not statistically significant (Table 3). However, the aIRRs for AA/AD were increased for FQs vs both amoxicillin [aIRR 1.50 (95% CI 1.01, 2.25)] and azithromycin [aIRR 2.15 (95% CI 1.27, 3.64)]. Mortality risks were significantly increased with FQs vs each of the five antibiotics of interest. Table S2 includes the aIRRs for FQs vs no antibiotics, other individual antibiotics, and multiple antibiotics concurrently. Although the point estimates varied slightly, the associations remained the same for each outcome when we evaluated FQs vs all antibiotics in aggregate (data not shown).

**TABLE 3:** Risk of adverse events with fluoroquinolones vs comparator antibiotics, 30‐d risk period

	Risk period for fluoroquinolone or comparator antibiotic			Unadjusted SCCSA model			Adjusted SCCSA model^a^	
	Number of Events	Number of person‐days	Rate of event/100 person‐days	IRR (95% CI)		*P* value	aIRR (95% CI)	*P* value
Ventricular Arrhythmia, N = 3154 patients with 3607 events^b^ and 47 900 observation periods								
Fluoroquinolone risk period	177	138 348	0.128	1.00			1.00	
Fluoroquinolone vs amoxicillin^c^	91	84 167	0.108	1.11 (0.86,1.44)		0.42	1.19 (0.91,1.54)	0.21
Fluoroquinolone vs azithromycin	55	47 580	0.116	1.02 (0.75,1.39)		0.91	1.10 (0.80,1.52)	0.54
Fluoroquinolone vs cefuroxime/cephalexin	52	42 151	0.123	1.00 (0.73,1.38)		0.99	1.07 (0.78,1.48)	0.68
Fluoroquinolone vs doxycycline	49	43 330	0.113	1.07 (0.77,1.48)		0.69	1.28 (0.92,1.78)	0.14
Fluoroquinolone vs SMX‐TMP	40	32 662	0.122	0.98 (0.69,1.39)		0.89	0.98 (0.68,1.39)	0.89
Aortic aneurysm and/or dissection, N = 2027 patients with 2187 events^b^ and 26 771 observation periods								
Fluoroquinolone risk period	124	88 606	0.140	1.00			1.00	
Fluoroquinolone vs amoxicillin^c^	32	37 586	0.085	1.56 (1.04,2.32)		0.03	1.50 (1.01,2.25)	0.046
Fluoroquinolone vs azithromycin	17	25 326	0.067	1.98 (1.18,3.33)		0.01	2.15 (1.27,3.64)	0.004
Fluoroquinolone vs cefuroxime/cephalexin	18	20 825	0.086	1.49 (0.90,2.48)		0.12	1.35 (0.81,2.24)	0.25
Fluoroquinolone vs doxycycline	13	18 218	0.071	1.76 (0.98,3.16)		0.06	1.81 (1.00,3.25)	0.05
Fluoroquinolone vs SMX‐TMP	27	18 849	0.143	0.90 (0.59,1.38)		0.63	0.81 (0.53,1.25)	0.34
Acute myocardial infarction, N = 13 504 patients with 14 899 events^b^ and 192 314 observation periods								
Fluoroquinolone risk period	672	580 518	0.116	1.00			1.00	
Fluoroquinolone vs amoxicillin^c^	314	311 422	0.101	1.03 (0.89,1.18)		0.72	1.01 (0.88,1.16)	0.91
Fluoroquinolone vs azithromycin	193	195 345	0.099	1.03 (0.88,1.22)		0.69	1.09 (0.93,1.29)	0.29
Fluoroquinolone vs cefuroxime/cephalexin	143	153 919	0.093	1.15 (0.96,1.38)		0.14	1.09 (0.91,1.31)	0.36
Fluoroquinolone vs doxycycline	141	148 150	0.095	1.09 (0.91,1.31)		0.36	1.16 (0.96,1.40)	0.12
Fluoroquinolone vs SMX‐TMP	105	128 214	0.082	1.25 (1.02,1.55)	0.04		1.17 (0.95,1.44)	0.15
Mortality, N = 109 024^d^ patients with 109 024 events^b^ and 1092 718 observation periods								
Fluoroquinolone risk period	7145	4 315 403	0.166	1.00			1.00	
Fluoroquinolone vs amoxicillin^c^	1360	1 368 299	0.099	1.29 (1.21,1.37)	<0.001		1.23 (1.16,1.31)	<0.001
Fluoroquinolone vs azithromycin	634	874 027	0.073	1.81 (1.67,1.97)	<0.001		1.99 (1.83,2.16)	<0.001
Fluoroquinolone vs cefuroxime/cephalexin	648	752 188	0.086	1.48 (1.36,1.61)	<0.001		1.29 (1.19,1.41)	<0.001
Fluoroquinolone vs doxycycline	649	639 450	0.101	1.21 (1.11,1.31)	<0.001		1.17 (1.08,1.28)	<0.001
Fluoroquinolone vs SMX‐TMP	663	741 697	0.089	1.47 (1.36,1.60)	<0.001		1.34 (1.23,1.45)	<0.001

Note

IRR, incidence rate ratio; SCCSA, self‐controlled case series analysis; SMX‐TMP, sulfamethoxazole‐trimethoprim.

^a^ Adjusted for time‐varying covariates of age, fiscal year, and respiratory, urinary, and skin and soft‐tissue infections.

^b^ Rows for fluoroquinolones vs “no antibiotics,” “other antibiotics,” and “multiple antibiotics” were removed so the sum of the events does not equal the total listed for each outcome (full results in Table S2).

^c^ The numbers in the rows that follow “fluoroquinolone risk period” are for the comparator antibiotics (eg amoxicillin, azithromycin).

^d^ N = 56 patients were removed due to only one observation period.

In a sensitivity analysis where patients with more than one of the same outcome were removed, the results were unchanged, except the IRR for AA/AD during FQ vs amoxicillin risk periods [aIRR 1.44 (95% CI 0.92, 2.24)] was no longer significant (Table S3). When the risk period was decreased to 10 days in a sensitivity analysis, the aIRRs for mortality remained significantly elevated with FQs vs each of the comparator antibiotics of interest (Supplementary table 4). Using a 60‐day risk period for the outcome of AA/AD, the increased incidence with FQs vs azithromycin remained significantly elevated [aIRR 1.96 (95%CI 1.29,3.00)], and again, the IRR was not significantly increased during FQ vsersus amoxicillin risk periods [aIRR 1.16 (95%CI 0.85,1.57)] (Supplementary Table 4). Finally, there was an increased aIRR for AMI with FQs vs doxycycline in both sensitivity analyses (Supplementary tables 3 and 4).

## 4 DISCUSSION

VHA is one of the largest integrated health systems in the United States, with large databases that provide the ideal mechanism to study rare, but serious adverse drug reactions that were not identified during pre‐marketing trials. Our findings in Veterans suggest an increased incidence of AA/AD with the FQs vs both amoxicillin and azithromycin. We also found an increased incidence of 30‐day, all‐cause mortality with the FQs vs each comparator antibiotic of interest. The SCCSA automatically controls for both known and unknown time‐invariant confounders as patients serve as their own controls. In addition, this removes any bias that may be introduced in the selection of controls. Finally, we also included potential time‐varying confounders in the model to try to limit residual confounding due to differences between patients who receive FQs vs other antibiotics.

Our results regarding a positive association between FQ use and AA/AD corroborate prior reports, despite different study methods.^11^, ^12^, ^13^, ^15^ Lee et al used a case‐crossover design and found increased odds of exposure to FQs during the hazard period (60 days prior to AA/AD event) vs the referent period (one of three randomly selected 60‐day periods between 120 and 300 days prior to AA/AD event) (OR 2.15; 95%CI 1.14‐6.46).^15^ Two additional studies evaluated the risk of AA/AD with FQ exposure vs non‐exposure periods, and the conclusions were the same.^11^, ^12^ However, these results provide no information about the risk with FQs vs other antibiotics. This is important because the provider must decide which antibiotic, among those available, is most appropriate for a patient, and this decision involves consideration of antibiotic side effect profiles. Pasternak et al found an increased hazard of AA/AD with FQs vs amoxicillin (HR 1.66; 95% CI 1.12‐2.46) using a propensity score matched cohort^13^; however, we evaluated other antibiotics in addition to amoxicillin.

The data regarding a potential association between FQs and overall mortality are conflicting. In a meta‐analysis by Liu and colleagues that included 11 studies, an increased risk of overall mortality was not found (RR 1.02; 95%CI 0.76‐1.37).^16^ Although five of the studies had point estimates >1, the only study with a significant positive association was conducted in Veterans by Rao et al.^9^ They observed a higher risk of all‐cause mortality with levofloxacin vs amoxicillin and azithromycin at both days 1‐5 and 6‐10 (HR levofloxacin vs amoxicillin days 1‐5:2.49; 95%CI 1.7‐3.64 and days 6‐10:1.95; 95%CI 1.32‐2.88 and HR levofloxacin vs azithromycin days 1‐5:1.68; 95%CI 1.15‐2.47 and days 6‐10:1.71; 95%CI 1.15‐2.55).^9^ Our findings of increased all‐cause mortality with FQs vs each of the antibiotics in the study remained significant with a 10‐day risk period and adds to the literature. However, we cannot state the FQ was the proximate cause of a fatal event, as patients may have been more seriously ill during the times when they received a FQ vs other antibiotics even though all were outpatients. The increased risk of death merits attention and should provide further impetus for prescribers to carefully consider their antibiotic choice.

We did not find an increased risk of AMI or VenA with FQs vs the comparator antibiotics, which is consistent with some of the literature; although, data are limited. A recent meta‐analysis of AMI in FQ users vs non‐users found a small increased risk (OR 1.18; 95%CI 1.00‐1.38).^17^ However, a large study of Medicare beneficiaries, that was not part of the meta‐analysis, did not find an association between levofloxacin and AMI after adjusting for a wide range of potential confounders.^10^ For the outcome of VenA, results published in the literature comparing FQs with other antibiotics have also been mixed.^8^, ^9^, ^18^, ^19^ In the previously mentioned cohort study in Veterans by Rao et al, the authors found an increased hazard of serious VenA with levofloxacin vs amoxicillin at treatment days 1‐5 (HR 2.43; 95%CI 1.56‐3.79) and 6‐10 (HR 1.75; 95%CI 1.09‐2.82).^9^ Chou and colleagues conducted a similar study using the Taiwan National Health Insurance database and found increased odds of VenA with FQs as a group vs amoxicillin‐clavulanate (aOR 2.07; 95%CI 1.56‐2.76); however, when they evaluated the FQs individually, only moxifloxacin was associated with increased odds of VenA compared with amoxicillin‐clavulanate (aOR 3.3; 95%CI 2.07‐5.25).^8^ In another study of national data from Korea, similar findings were observed; namely, only moxifloxacin was associated with increased odds of VenA compared with cefixime (aOR 1.87; 95%CI 1.15‐3.11).^18^ Conversely, Inghammar et al used propensity score matching with many variables and found no increased incidence of serious arrhythmias with FQs vs penicillin VK (RR 0.85; 95%CI 0.61‐1.18).^19^ Differences among the results of these studies, including ours, may be due to varying patient population size and characteristics or residual confounding.

Our findings have clinical implications. The results of our study support the FDA’s recommendation that FQs should be avoided in patients with risk factors for AA/AD unless there are no viable alternatives.^4^ These risk factors include smoking, advanced age, male sex, hypertension, and atherosclerosis. Despite the evidence, a recent paper found that 20% of patients with known AA received FQs during a hospitalization before the repair, suggesting providers were unaware or unconvinced of the potential risk.^20^ Also, the potential increased risk of all‐cause mortality with the FQs vs other antibiotics supports recommendations to limit FQ use. However, given these recommendations, providers may prescribe alternative antibiotics that have other serious adverse effects. For example, sulfamethoxazole‐trimethoprim has been associated with hyperkalemia and renal failure, especially in elderly patients and those taking other medications that can raise serum potassium.^21^

Despite the strengths of our design, limitations remain that are inherent with observational studies. Although fixed confounders are controlled for in an SCCSA, and we included important time‐varying covariates, residual time‐varying confounding is still possible. We adjusted for age, fiscal year, and respiratory, urinary, and skin and soft‐tissue infections, but could not measure severity of infection. While our study included only outpatients, FQs may have been preferentially used over other antibiotics in patients with more severe illness. Also, we did not evaluate the risk of VenA, AMI, AA/AD, and all‐cause mortality with the FQs individually, so results may differ among the antibiotics in that class. Finally, our study population was predominantly elderly men, so the findings may not be fully generalizable to other populations.

## 5 CONCLUSION

We found that FQs were associated with an increased risk of AA/AD vs both amoxicillin and azithromycin and an increased risk of all‐cause mortality vs many antibiotics commonly used for outpatient infections. Although the differences in event rates are small, FQs should be reserved for serious infections where there are no suitable alternatives.

## 6 DATA SHARING AND DATA ACCESSIBILITY

The data that support the findings of this study are available on request from the corresponding author. The data are not publicly available due to privacy or ethical restrictions.

## DISCLOSURE

The authors have no potential conflicts of interest.

## AUTHORS' CONTRIBUTIONS

1. Have made substantial contributions to conception and design (SA, NS, XZ, RZ, DD, KE, PG, MG, DM, FC), or acquisition of data (XZ, RZ, DD) or analysis (XZ, RZ, DD) and interpretation of data (SA, NS, XZ, RZ, DD, KE, PG, MG, DM, FC); and
2. Been involved in drafting the manuscript (SA, XZ, FC) or revising it critically for important intellectual content (NS, RZ, DD, KE, PG, MG, DM); and
3. Given final approval of the version to be published (SA, NS, XZ, RZ, DD, KE, PG, MG, DM, FC).
4. Each author should have participated sufficiently in the work to take public responsibility for appropriate portions of the content; and agreed to be accountable for all aspects of the work in ensuring that questions related to the accuracy or integrity of any part of the work are appropriately investigated and resolved. (SA, NS, XZ, RZ, DD, KE, PG, MG, DM, FC)

## Supporting information

Table S1‐S4
