# Supplementary material for: Serious cardiovascular adverse events with fluoroquinolones versus other antibiotics: A self‐controlled case series analysis
Source: Pharmacol Res Perspect. 2020 Oct 12;8(6):e00664. doi: 10.1002/prp2.664 (PMC7550792; doi:10.1002/prp2.664)
Supplement: Supplementary file 1 — Table S1‐S4 [file PRP2-8-e00664-s001.docx]

**Supplementary Table 1. ICD-9-CM and ICD-10-CM Codes for Outcomes of Ventricular Arrhythmia, Aortic Aneurysm/Dissection, and Acute Myocardial Infarction and Covariates of Infection**

| ***Outcomes*** | | **ICD-9-CM Diagnosis Codes** | **ICD-10-CM Diagnosis Codes** |
| --- | --- | --- | --- |
| **Ventricular Arrhythmia** | **Ventricular tachycardia** | 427.1 Paroxysmal ventricular tachycardia  427.0 Paroxysmal supraventricular tachycardia (Not SVT) | I47.0 Re-entry ventricular arrhythmia  I47.1 Supraventricular tachycardia (Not SVT) I47.2 Ventricular tachycardia |
|  | **Ventricular flutter** | 427.42 Ventricular flutter | I49.02 Ventricular flutter |
|  | **Ventricular fibrillation** | 427.41 Ventricular fibrillation | I49.01 Ventricular fibrillation |
|  | **Torsades de pointes** | 426.82 Long QT syndrome | I45.81 Long QT syndrome |
| **Aortic Aneurysm/ Dissection** | **Aortic aneurysm** | 441.1 Thoracic aneurysm, ruptured  441.2 Thoracic aneurysm without mention of rupture  441.3 Abdominal aneurysm, ruptured  441.4 Abdominal aneurysm without mention of rupture  441.5 Aortic aneurysm of unspecified site, ruptured  441.6 Thoracoabdominal aneurysm, ruptured  441.7 Thoracoabdominal aneurysm, without mention of rupture  441.9 Aortic aneurysm of unspecified site without mention of rupture | I71.1 Thoracic aortic aneurysm, ruptured I71.2 Thoracic aortic aneurysm, without rupture I71.3 Abdominal aortic aneurysm, ruptured I71.4 Abdominal aortic aneurysm, without rupture I71.5 Thoracoabdominal aortic aneurysm, ruptured I71.6 Thoracoabdominal aortic aneurysm, without rupture I71.8 Aortic aneurysm of unspecified site, ruptured I71.9 Aortic aneurysm of unspecified site, without rupture |
|  | **Aortic dissection** | 441.0 Dissection of aorta  441.00 Dissection of aorta, unspecified site 441.01 Dissection of aorta, thoracic  441.02 Dissection of aorta, abdominal  441.03 Dissection of aorta, thoracoabdominal | I71.00 Dissection of unspecified site of aorta I71.01 Dissection of thoracic aorta I71.02 Dissection of abdominal aorta I71.03 Dissection of thoracoabdominal aorta |

| ***Outcomes (continued)*** | | **ICD-9-CM Diagnosis Codes** | **ICD-10-CM Diagnosis Codes** |
| --- | --- | --- | --- |
| **Acute Myocardial Infarction** |  | 410.xx Acute myocardial infarction | I21.0 ST elevation (STEMI) myocardial infarction of anterior wall  I21.1 ST elevation (STEMI) myocardial infarction of inferior wall I21.2 ST elevation (STEMI) myocardial infarction of other sites I21.3 ST elevation (STEMI) myocardial infarction of unspecified site I21.4 Non-ST elevation (NSTEMI) myocardial infarction I21.9 Acute myocardial infarction, unspecified |
| ***Covariates of Infection*** | | **ICD-9-CM Diagnosis Codes** | **ICD-10-CM Diagnosis Codes** |
| **Respiratory Tract** | **Pneumonia** | 481 Pneumococcal pneumonia (Streptococcus pneumoniae pneumonia) 482.xx Other bacterial pneumonia 483.x Pneumonia due to other specified organism  485 Bronchopneumonia, organism unspecified  486 Pneumonia, organism unspecified | J69.x Pneumonitis due to inhalation of food and vomit  J13 Pneumonia due to Streptococcus pneumoniae J14 Pneumonia due to Hemophilus influenzae J15.xxx Bacterial pneumonia, not elsewhere classified J16.x Pneumonia due to other infectious organisms, not elsewhere classified J17 Pneumonia in diseases classified elsewhere J18.x Pneumonia, unspecified organism |
|  | **Chronic obstructive pulmonary disease exacerbation** | 491 Chronic bronchitis 491.0 Simple chronic bronchitis 491.1 Mucopurulent chronic bronchitis 491.2 Obstructive chronic bronchitis 491.20 Obstructive chronic bronchitis without exacerbation 491.21 Acute exacerbation of chronic obstructive pulmonary disease 491.22 Obstructive chronic bronchitis with acute bronchitis 491.8 Other chronic bronchitis 491.9 Unspecified chronic bronchitis 492 Emphysema 492.0 Emphysematous bleb 492.8 Other emphysema 494 Bronchiectasis 494.0 Bronchiectasis without acute exacerbation 494.1 Bronchiectasis with acute exacerbation 496 Chronic airway obstruction, not elsewhere classified | J40 Bronchitis, not specified as acute or chronic J41.0 Simple chronic bronchitis J41.1 Mucopurulent chronic bronchitis J41.8 Mixed simple and mucopurulent chronic bronchitis J42 Unspecified chronic bronchitis J43 Emphysema J44.x Other chronic obstructive pulmonary disease J47.x Bronchiectasis |
|  | **Bronchitis** | 466.0 Acute bronchitis  466.1 Acute bronchiolitis  490 Bronchitis, not specified as acute or chronic 491.xx Chronic bronchitis | J20.0 Acute bronchitis due to Mycoplasma pneumoniae  J20.1 Acute bronchitis due to Hemophilus influenzae  J20.2 Acute bronchitis due to streptococcus  J20.9 Acute bronchitis, unspecified J21.9 Acute bronchiolitis  J40 Bronchitis, not specified as acute or chronic  J41.x Simple and mucopurulent chronic bronchitis  J42 Unspecified chronic bronchitis |
|  | **Pharyngitis** | 462 Acute pharyngitis  472.1 Chronic pharyngitis  472.2 Chronic nasopharyngitis | J31.1 Chronic nasopharyngitis J31.2 Chronic pharyngitis J02 Acute pharyngitis |
|  | **Sinusitis** | 461.x Acute sinusitis  473.x Chronic sinusitis  478.19 Other disease of nasal cavity and sinuses | J32.x Chronic sinusitis J01.xx Acute sinusitis J34.1 Cyst and mucocele of nose and nasal sinus J34.89 Other specified disorders of nose and nasal sinuses |
|  | **Cough** | 033.0 Whooping cough  786.2 Cough | A37.xx Whooping cough  R05 Cough |
|  | **Upper respiratory infection** | 460 Acute nasopharyngitis  461.x Acute sinusitis 462 Acute pharyngitis 463 Acute tonsillitis 464.xx Acute laryngitis and tracheitis 465.x Acute upper respiratory infections of multiple or unspecified sites | J00 Acute nasopharyngitis [common cold]  J01.xx Acute sinusitis  J02.x Acute pharyngitis  J03.xx Acute tonsillitis  J04.xx Acute laryngitis and tracheitis  J05.xx Acute obstructive laryngitis [croup] and epiglottitis  J06.x Acute upper respiratory infections of multiple and unspecified sites |
| **Urinary Tract** | **Urinary tract infection** | 590.9 infection of kidney, unspecified  599.0 UTI, site not specified  595.0 Acute cystitis  595.9 Cystitis, unspecified  788.1 Dysuria | N39.0 UTI, site not specified  R30.0 Acute cystitis  R30.9 Painful micturition, unspecified |
|  | **Pyelonephritis** | 590.0 Chronic pyelonephritis  590.1 Acute pyelonephritis  590.8 Other pyelonephritis or pyonephrosis not specified as acute or chronic | N10 Acute pyelonephritis N11.0 Nonobstructive reflux-associated chronic pyelonephritis N11.1 Chronic obstructive pyelonephritis |
|  | **Prostatitis** | 601.0 Acute prostatitis  601.1 Chronic prostatitis  601.9 Prostatitis, unspecified | N41.0 Acute prostatitis  N41.1 Chronic prostatitis  N41.9 Inflammatory disease of prostate, unspecified |
|  | **Bacteriuria** | **-** | R82.71 Bacteriuria |
| **Skin and Soft Tissue** | **Cellulitis** | 681.xx Cellulitis and abscess of finger and toe 682.x Other cellulitis and abscess | L03.01 Cellulitis of finger L03.03 Cellulitis of toe L03.11 Cellulitis of other parts of limb L03.211 Cellulitis of face L03.221 Cellulitis of neck L03.31 Cellulitis of trunk L03.81 Cellulitis of other sites L03.90 Cellulitis, unspecified |
|  | **Skin abscess** | 681.xx Cellulitis and abscess of finger and toe 682.x Other cellulitis and abscess | L02.01 Cutaneous abscess of face L02.11 Cutaneous abscess of neck L02.21 Cutaneous abscess of trunk L02.31 Cutaneous abscess of buttock L02.41 Cutaneous abscess of limb L02.51 Cutaneous abscess of hand L02.61 Cutaneous abscess of foot L02.81 Cutaneous abscess of other sites L02.91 Cutaneous abscess, unspecified |
|  | **Diabetic foot infection** | 250.80 Diabetes with other specified manifestations, type II or unspecified type, not stated as uncontrolled 250.81 Diabetes with other specified manifestations, type I (juvenile type), not stated as uncontrolled | E08.621 Diabetes mellitus due to underlying condition with foot ulcer E10.621 Type 1 diabetes mellitus with foot ulcer E11.621Type 2 diabetes mellitus with foot ulcer E13.621 Other specified diabetes mellitus with foot ulcer |
|  | **Skin and soft tissue infection** | 680.x Carbuncle and furuncle 681.xx Cellulitis and abscess of finger and toe 682.x Other cellulitis and abscess 683 Acute lymphadenitis 684 Impetigo 686.x Other local infections of skin and subcutaneous tissue | L00 Staphylococcal scalded skin syndrome L01 Impetigo L02.xxx Cutaneous abscess, furuncle and carbuncle L03.xxx Cellulitis and acute lymphangitis L04.x Acute lymphadenitis L08.xx Other local infections of skin and subcutaneous tissue |

ICD-9/10-CM=International Classification of Diseases, Ninth and Tenth Revisions, Clinical Modification

**Supplementary Table 2. Risk of Adverse Events with Fluoroquinolones Versus Comparator Antibiotics, 30-day risk period, *results added***

***for fluoroquinolones versus no antibiotics, other antibiotics, and multiple antibiotics***

|  | **Risk Period for Fluoroquinolone or Comparator Antibiotic** | | | **Unadjusted SCCSA model** | | | **Adjusted SCCSA model^a^** | | |
| --- | --- | --- | --- | --- | --- | --- | --- | --- | --- |
|  | **Number of Events** | **Number of person-Days** | **Rate of Event/100 person-days** | **IRR (95% CI)** | | **P value** | **aIRR (95% CI)** | **P value** | |
| ***Ventricular Arrhythmia,*** *N=3,154 patients with 3,607 events and 47,900 observation periods* | | | | | | | | | |
| Fluoroquinolone Risk Period | 177 | 138348 | 0.128 | 1.00 | |  | 1.00 |  | |
| Fluoroquinolone vs. Amoxicillin^b^ | 91 | 84167 | 0.108 | 1.11 (0.86,1.44) | | 0.42 | 1.19 (0.91,1.54) | 0.21 | |
| Fluoroquinolone vs. Azithromycin | 55 | 47580 | 0.116 | 1.02 (0.75,1.39) | | 0.91 | 1.10 (0.80,1.52) | 0.54 | |
| Fluoroquinolone vs. Cefuroxime/Cephalexin | 52 | 42151 | 0.123 | 1.00 (0.73,1.38) | | 0.99 | 1.07 (0.78,1.48) | 0.68 | |
| Fluoroquinolone vs. Doxycycline | 49 | 43330 | 0.113 | 1.07 (0.77,1.48) | | 0.69 | 1.28 (0.92,1.78) | 0.14 | |
| Fluoroquinolone vs.  SMX-TMP | 40 | 32662 | 0.122 | 0.98 (0.69,1.39) | | 0.89 | 0.98 (0.68,1.39) | 0.89 | |
| Fluoroquinolone vs. Other Antibiotics | 47 | 49775 | 0.094 | 1.31 (0.94,1.82) | | 0.11 | 1.35 (0.97,1.88) | 0.08 | |
| Fluoroquinolone vs. Multiple Antibiotics | 99 | 68516 | 0.144 | 0.87 (0.67,1.11) | | 0.26 | 0.94 (0.72,1.21) | 0.61 | |
| Fluoroquinolone vs.  No Antibiotics | 2997 | 4663010 | 0.064 | 1.85 (1.59,2.17) | | <0.001 | 1.54 (1.30,1.83) | <0.001 | |
| ***Aortic Aneurysm and/or Dissection,*** *N=2,027 patients with 2,187 events and 26,771 observation periods* | | | | | | | | | |
| Fluoroquinolone Risk Period | 124 | 88606 | 0.140 | 1.00 | |  | 1.00 |  | |
| Fluoroquinolone vs. Amoxicillin^b^ | 32 | 37586 | 0.085 | 1.56 (1.04,2.32) | | 0.03 | 1.50 (1.01,2.25) | 0.046 | |
| Fluoroquinolone vs. Azithromycin | 17 | 25326 | 0.067 | 1.98 (1.18,3.33) | | 0.01 | 2.15 (1.27,3.64) | 0.004 | |
| Fluoroquinolone vs. Cefuroxime/Cephalexin | 18 | 20825 | 0.086 | 1.49 (0.90,2.48) | | 0.12 | 1.35 (0.81,2.24) | 0.25 | |
| Fluoroquinolone vs. Doxycycline | 13 | 18218 | 0.071 | 1.76 (0.98,3.16) | | 0.06 | 1.81 (1.00,3.25) | 0.05 | |
| Fluoroquinolone vs.  SMX-TMP | 27 | 18849 | 0.143 | 0.90 (0.59,1.38) | | 0.63 | 0.81 (0.53,1.25) | 0.34 | |
| Fluoroquinolone vs. Other Antibiotics | 29 | 22528 | 0.129 | 1.03 (0.68,1.56) | | 0.90 | 0.92 (0.61,1.40) | 0.71 | |
| Fluoroquinolone vs. Multiple Antibiotics | 53 | 32789 | 0.162 | 0.83 (0.60,1.15) | | 0.27 | 0.81 (0.58,1.13) | 0.22 | |
| Fluoroquinolone vs.  No Antibiotics | 1874 | 3118956 | 0.060 | 2.22 (1.84,2.67) | | <0.001 | 1.64 (1.33,2.03) | <0.001 | |
| ***Acute Myocardial Infarction,*** *N=13,504 patients with 14,899 events and 192,314 observation periods* | | | | | | | | | |
| Fluoroquinolone Risk Period | 672 | 580518 | 0.116 | 1.00 | |  | 1.00 |  | |
| Fluoroquinolone vs. Amoxicillin^b^ | 314 | 311422 | 0.101 | 1.03 (0.89,1.18) | | 0.72 | 1.01 (0.88,1.16) | 0.91 | |
| Fluoroquinolone vs. Azithromycin | 193 | 195345 | 0.099 | 1.03 (0.88,1.22) | | 0.69 | 1.09 (0.93,1.29) | 0.29 | |
| Fluoroquinolone vs. Cefuroxime/Cephalexin | 143 | 153919 | 0.093 | 1.15 (0.96,1.38) | | 0.14 | 1.09 (0.91,1.31) | 0.36 | |
| Fluoroquinolone vs. Doxycycline | 141 | 148150 | 0.095 | 1.09 (0.91,1.31) | | 0.36 | 1.16 (0.96,1.40) | 0.12 | |
| Fluoroquinolone vs.  SMX-TMP | 105 | 128214 | 0.082 | 1.25 (1.02,1.55) | 0.04 | | 1.17 (0.95,1.44) | | 0.15 |
| Fluoroquinolone vs. Other Antibiotics | 202 | 201199 | 0.100 | 1.05 (0.89,1.23) | 0.57 | | 0.99 (0.84,1.16) | | 0.88 |
| Fluoroquinolone vs. Multiple Antibiotics | 334 | 277643 | 0.120 | 0.92 (0.81,1.06) | 0.24 | | 0.94 (0.82,1.08) | | 0.39 |
| Fluoroquinolone vs.  No Antibiotics | 12795 | 19491873 | 0.066 | 1.56 (1.44,1.69) | <0.001 | | 1.24 (1.14,1.36) | | <0.001 |
| ***Mortality,*** *N=109,024^c^ patients with 109,024 events and 1,092,718 observation periods* | | | | | | | | | |
| Fluoroquinolone Risk Period | 7145 | 4315403 | 0.166 | 1.00 |  | | 1.00 | |  |
| Fluoroquinolone vs. Amoxicillin^b^ | 1360 | 1368299 | 0.099 | 1.29 (1.21,1.37) | <0.001 | | 1.23 (1.16,1.31) | | <0.001 |
| Fluoroquinolone vs. Azithromycin | 634 | 874027 | 0.073 | 1.81 (1.67,1.97) | <0.001 | | 1.99 (1.83,2.16) | | <0.001 |
| Fluoroquinolone vs. Cefuroxime/Cephalexin | 648 | 752188 | 0.086 | 1.48 (1.36,1.61) | <0.001 | | 1.29 (1.19,1.41) | | <0.001 |
| Fluoroquinolone vs. Doxycycline | 649 | 639450 | 0.101 | 1.21 (1.11,1.31) | <0.001 | | 1.17 (1.08,1.28) | | <0.001 |
| Fluoroquinolone vs.  SMX-TMP | 663 | 741697 | 0.089 | 1.47 (1.36,1.60) | <0.001 | | 1.34 (1.23,1.45) | | <0.001 |
| Fluoroquinolone vs. Other Antibiotics | 1040 | 939127 | 0.111 | 1.17 (1.09,1.25) | <0.001 | | 1.04 (0.97,1.12) | | 0.23 |
| Fluoroquinolone vs. Multiple Antibiotics | 2449 | 1487788 | 0.165 | 0.94 (0.89,0.98) | 0.007 | | 0.92 (0.88,0.97) | | 0.001 |
| Fluoroquinolone vs.  No Antibiotics | 94436 | 104391205 | 0.090 | 1.35 (1.31,1.38) | <0.001 | | 1.06 (1.03,1.09) | | <0.001 |

SCCSA=self-controlled case series analysis; IRR= incidence rate ratio; SMX-TMP=sulfamethoxazole-trimethoprim

^a^Adjusted for time-varying covariates of age, fiscal year, and respiratory, urinary, and skin and soft-tissue infections

^b^The numbers in the rows that follow “fluoroquinolone risk period” are for the comparator antibiotics (e.g., amoxicillin, azithromycin).

^c^N=56 patients were removed due to only one observation period

**Supplementary Table 3. Risk of Adverse Events with Fluoroquinolones Versus Comparator Antibiotics, 30-day risk period,**

***patients with multiple outcomes removed***

|  | **Risk Period for Fluoroquinolone or Comparator Antibiotic** | | | **Unadjusted SCCSA model** | | | **Adjusted SCCSA model**^a^ | |
| --- | --- | --- | --- | --- | --- | --- | --- | --- |
|  | **Number of Events** | **Number of Risk Days** | **Rate of Event/100 person-days** | **IRR (95% CI)** | | **P value** | **aIRR (95% CI)** | **P value** |
| ***Ventricular Arrhythmia****, N=2,797 (88.7%) patients with 2,797 events and 41,587 (86.8%) observation periods* | | | | | | | | |
| Fluoroquinolone Risk Period | 130 | 121870 | 0.107 | 1.00 | |  | 1.00 |  |
| Fluoroquinolone vs. Amoxicillin^b^ | 70 | 72479 | 0.097 | 1.00 (0.74,1.35) | | 0.99 | 1.07 (0.79,1.45) | 0.66 |
| Fluoroquinolone vs. Azithromycin | 39 | 41162 | 0.095 | 0.98 (0.68,1.42) | | 0.92 | 1.09 (0.75,1.59) | 0.65 |
| Fluoroquinolone vs. Cefuroxime/Cephalexin | 34 | 35321 | 0.096 | 1.01 (0.69,1.49) | | 0.95 | 1.08 (0.73,1.59) | 0.71 |
| Fluoroquinolone vs. Doxycycline | 28 | 36320 | 0.077 | 1.25 (0.82,1.89) | | 0.30 | 1.52(1.00,2.32) | 0.05 |
| Fluoroquinolone vs.  SMX-TMP | 26 | 28546 | 0.091 | 1.06 (0.69,1.63) | | 0.78 | 1.07 (0.69,1.65) | 0.77 |
| Fluoroquinolone vs. Other Antibiotics | 32 | 42418 | 0.075 | 1.31 (0.88,1.94) | | 0.18 | 1.33 (0.89,1.99) | 0.16 |
| Fluoroquinolone vs. Multiple Antibiotics | 78 | 58552 | 0.133 | 0.74 (0.56,0.99) | | 0.04 | 0.81 (0.60,1.09) | 0.16 |
| Fluoroquinolone vs.  No Antibiotics | 2360 | 4127529 | 0.057 | 1.71 (1.42,2.04) | | <0.001 | 1.44 (1.18,1.76) | <0.001 |
| ***Aortic Aneurysm and/or Dissection,*** *N=1,887 (93.1%) patients with 1,887 events and 24,816 (92.7%) observation periods* | | | | | | | | |
| Fluoroquinolone Risk Period | 98 | 82276 | 0.119 | 1.00 | |  | 1.00 |  |
| Fluoroquinolone vs. Amoxicillin^b^ | 26 | 34328 | 0.076 | 1.45 (0.93,2.26) | | 0.10 | 1.44 (0.92,2.24) | 0.11 |
| Fluoroquinolone vs. Azithromycin | 14 | 23407 | 0.060 | 1.90 (1.07,3.38) | | 0.03 | 2.11 (1.18,3.77) | 0.01 |
| Fluoroquinolone vs. Cefuroxime/Cephalexin | 13 | 19484 | 0.067 | 1.67 (0.92,3.01) | | 0.09 | 1.54 (0.85,2.78) | 0.16 |
| Fluoroquinolone vs. Doxycycline | 12 | 17150 | 0.070 | 1.55 (0.84,2.85) | | 0.16 | 1.63 (0.88,3.01) | 0.12 |
| Fluoroquinolone vs.  SMX-TMP | 23 | 17310 | 0.133 | 0.81 (0.51,1.29) | | 0.38 | 0.73 (0.46,1.17) | 0.19 |
| Fluoroquinolone vs. Other Antibiotics | 23 | 20557 | 0.112 | 0.98 (0.62,1.57) | | 0.94 | 0.91 (0.57,1.45) | 0.69 |
| Fluoroquinolone vs. Multiple Antibiotics | 38 | 30334 | 0.125 | 0.91 (0.62,1.34) | | 0.63 | 0.90 (0.61,1.32) | 0.59 |
| Fluoroquinolone vs.  No Antibiotics | 1640 | 2907696 | 0.056 | 1.99 (1.62,2.46) | | <0.001 | 1.54 (1.22,1.94) | <0.001 |
| ***Acute Myocardial Infarction****,* *N=12,357 (91.5%) patients with 12,357 events and 174,247 (90.6%) observation periods* | | | | | | | | |
| Fluoroquinolone Risk Period | 535 | 527025 | 0.102 | 1.00 | |  | 1.00 |  |
| Fluoroquinolone vs. Amoxicillin^b^ | 235 | 280145 | 0.084 | 1.06 (0.91,1.25) | | 0.44 | 1.05 (0.89,1.23) | 0.57 |
| Fluoroquinolone vs. Azithromycin | 156 | 175777 | 0.089 | 1.00 (0.83,1.20) | | 0.99 | 1.06 (0.88,1.28) | 0.52 |
| Fluoroquinolone vs. Cefuroxime/Cephalexin | 103 | 137081 | 0.075 | 1.22 (0.98,1.51) | | 0.08 | 1.16 (0.93,1.44) | 0.18 |
| Fluoroquinolone vs. Doxycycline | 98 | 132792 | 0.074 | 1.21 (0.97,1.51) | | 0.09 | 1.29 (1.03,1.61) | 0.02 |
| Fluoroquinolone vs.  SMX-TMP | 81 | 117251 | 0.069 | 1.31 (1.03,1.66) | 0.03 | | 1.23 (0.97,1.57) | 0.09 |
| Fluoroquinolone vs. Other Antibiotics | 168 | 179936 | 0.093 | 0.97 (0.81,1.16) | 0.74 | | 0.92 (0.77,1.10) | 0.36 |
| Fluoroquinolone vs. Multiple Antibiotics | 266 | 249943 | 0.106 | 0.91 (0.78,1.05) | 0.20 | | 0.92 (0.79,1.08) | 0.32 |
| Fluoroquinolone vs.  No Antibiotics | 10715 | 17892284 | 0.060 | 1.51 (1.38,1.65) | <0.001 | | 1.22 (1.10,1.35) | <0.001 |

SCCSA=self-controlled case series analysis; IRR= incidence rate ratio; SMX-TMP=sulfamethoxazole-trimethoprim

^a^Adjusted for time-varying covariates of age, fiscal year, and respiratory, urinary, and skin and soft-tissue infections

^b^The numbers in the rows that follow “fluoroquinolone risk period” are for the comparator antibiotics (e.g., amoxicillin, azithromycin).

**Supplementary Table 4. Risk of Adverse Events with Fluoroquinolones Versus Comparator Antibiotics,**

***10-day risk period for all outcomes and 60 days for Aortic Aneurysm/Dissection***

|  | **Risk Period for Fluoroquinolone or Comparator Antibiotic** | | | **Unadjusted SCCSA model** | | | **Adjusted SCCSA model**^a^ | | |
| --- | --- | --- | --- | --- | --- | --- | --- | --- | --- |
|  | **Number of Events** | **Number of Risk Days** | **Rate of Event/100 person-days** | **IRR (95% CI)** | | **P value** | **aIRR (95% CI)** | **P value** | |
| ***Ventricular Arrhythmia,*** *10-day risk period, N=3,154 patients with 3,589 events and 51,076 observation periods* | | | | | | | | | |
| Fluoroquinolone Risk Period | 92 | 56929 | 0.162 | 1.00 | |  | 1.00 |  | |
| Fluoroquinolone vs. Amoxicillin^b^ | 39 | 35105 | 0.111 | 1.40 (0.96,2.05) | | 0.08 | 1.42 (0.97,2.09) | 0.07 | |
| Fluoroquinolone vs. Azithromycin | 21 | 18709 | 0.112 | 1.35 (0.83,2.18) | | 0.23 | 1.39 (0.86,2.26) | 0.18 | |
| Fluoroquinolone vs. Cefuroxime/Cephalexin | 20 | 17008 | 0.118 | 1.36 (0.83,2.21) | | 0.22 | 1.41 (0.86,2.31) | 0.17 | |
| Fluoroquinolone vs. Doxycycline | 22 | 19289 | 0.114 | 1.36 (0.85,2.18) | | 0.21 | 1.54 (0.96,2.48) | 0.08 | |
| Fluoroquinolone vs.  SMX-TMP | 21 | 14619 | 0.144 | 1.07 (0.66,1.72) | | 0.79 | 1.10 (0.68,1.78) | 0.71 | |
| Fluoroquinolone vs.  Other Antibiotics | 26 | 21874 | 0.119 | 1.32 (0.85,2.05) | | 0.22 | 1.37 (0.88,2.13) | 0.17 | |
| Fluoroquinolone vs.  Multiple Antibiotics | 32 | 17313 | 0.185 | 0.86 (0.57,1.30) | | 0.48 | 0.92 (0.61,1.39) | 0.69 | |
| Fluoroquinolone vs.  No Antibiotics | 3316 | 4968693 | 0.067 | 2.22 (1.80,2.74) | | <0.001 | 1.71 (1.37,2.13) | <0.001 | |
| ***Aortic Aneurysm and/or Dissection*,** *10-day risk period, N=2,027 patients with 2,190 events and 28,469 observation periods* | | | | | | | | | |
| Fluoroquinolone Risk Period | 64 | 36495 | 0.175 | 1.00 | |  | 1.00 |  | |
| Fluoroquinolone vs. Amoxicillin^b^ | 14 | 15530 | 0.090 | 1.85 (1.03,3.33) | | 0.04 | 1.78 (0.99,3.21) | 0.05 | |
| Fluoroquinolone vs. Azithromycin | 9 | 9755 | 0.092 | 1.82 (0.90,3.70) | | 0.10 | 1.99 (0.98,4.06) | 0.06 | |
| Fluoroquinolone vs. Cefuroxime/Cephalexin | 7 | 8647 | 0.081 | 2.02 (0.92,4.44) | | 0.08 | 1.81 (0.82,4.00) | 0.14 | |
| Fluoroquinolone vs. Doxycycline | 10 | 8435 | 0.119 | 1.32 (0.67,2.60) | | 0.42 | 1.33 (0.67,2.62) | 0.42 | |
| Fluoroquinolone vs.  SMX-TMP | 15 | 8378 | 0.179 | 0.92 (0.52,1.63) | | 0.77 | 0.84 (0.47,1.50) | 0.56 | |
| Fluoroquinolone vs.  Other Antibiotics | 17 | 10025 | 0.170 | 1.00 (0.58,1.73) | | 0.99 | 0.90 (0.52,1.55) | 0.70 | |
| Fluoroquinolone vs.  Multiple Antibiotics | 13 | 8495 | 0.153 | 1.12 (0.61,2.05) | | 0.71 | 1.08 (0.59,1.98) | 0.81 | |
| Fluoroquinolone vs.  No Antibiotics | 2041 | 3277923 | 0.062 | 2.66 (2.06,3.42) | | <0.001 | 1.85 (1.41,2.43) | <0.001 | |
| ***Aortic Aneurysm and/or Dissection,*** *60-day risk period, N=2,027 patients with 2,188 events and 24,820 observation periods* | | | | | | | | | |
| Fluoroquinolone Risk Period | 192 | 155123 | 0.124 | 1.00 | |  | 1.00 |  | |
| Fluoroquinolone vs. Amoxicillin^b^ | 60 | 62594 | 0.096 | 1.19 (0.88,1.61) | | 0.26 | 1.16 (0.85,1.57) | 0.36 | |
| Fluoroquinolone vs. Azithromycin | 27 | 43428 | 0.062 | 1.89 (1.25,2.88) | | 0.003 | 1.96 (1.29,3.00) | 0.002 | |
| Fluoroquinolone vs. Cefuroxime/Cephalexin | 27 | 34861 | 0.077 | 1.46 (0.96,2.22) | | 0.07 | 1.37 (0.90,2.09) | 0.14 | |
| Fluoroquinolone vs. Doxycycline | 20 | 30061 | 0.067 | 1.66 (1.03,2.67) | | 0.04 | 1.68 (1.04,2.72) | 0.03 | |
| Fluoroquinolone vs.  SMX-TMP | 37 | 30213 | 0.122 | 0.92 (0.64,1.33) | | 0.67 | 0.87 (0.60,1.26) | 0.45 | |
| Fluoroquinolone vs.  Other Antibiotics | 35 | 36231 | 0.097 | 1.20 (0.83,1.74) | | 0.34 | 1.12 (0.77,1.64) | 0.54 | |
| Fluoroquinolone vs.  Multiple Antibiotics | 117 | 84804 | 0.138 | 0.84 (0.66,1.07) | | 0.15 | 0.83 (0.65,1.06) | 0.15 | |
| Fluoroquinolone vs.  No Antibiotics | 1673 | 2906368 | 0.058 | 2.07 (1.77,2.42) | | <0.001 | 1.74 (1.45,2.09) | <0.001 | |
| ***Acute Myocardial Infarction*,** *10-day risk period,* *N=13,504 patients with 14,871 events and 204,937 observation periods* | | | | | | | | | |
| Fluoroquinolone Risk Period | 363 | 241069 | 0.151 | 1.00 | |  | 1.00 |  | |
| Fluoroquinolone vs. Amoxicillin^b^ | 153 | 129034 | 0.119 | 1.15 (0.95,1.40) | | 0.15 | 1.12 (0.92,1.35) | 0.26 | |
| Fluoroquinolone vs. Azithromycin | 108 | 75596 | 0.143 | 0.94 (0.76,1.17) | | 0.60 | 0.99 (0.79,1.23) | 0.90 | |
| Fluoroquinolone vs. Cefuroxime/Cephalexin | 76 | 63007 | 0.121 | 1.16 (0.90,1.48) | | 0.26 | 1.09 (0.85,1.40) | 0.50 | |
| Fluoroquinolone vs. Doxycycline | 69 | 66229 | 0.104 | 1.31 (1.01,1.71) | | 0.04 | 1.36 (1.04,1.77) | 0.02 | |
| Fluoroquinolone vs.  SMX-TMP | 56 | 57896 | 0.097 | 1.39 (1.04,1.84) | 0.03 | | 1.30 (0.97,1.73) | | 0.07 |
| Fluoroquinolone vs.  Other Antibiotics | 113 | 89597 | 0.126 | 1.10 (0.89,1.37) | 0.37 | | 1.03 (0.83,1.28) | | 0.76 |
| Fluoroquinolone vs.  Multiple Antibiotics | 106 | 72437 | 0.146 | 1.01 (0.81,1.25) | 0.96 | | 1.00 (0.80,1.24) | | 0.98 |
| Fluoroquinolone vs.  No Antibiotics | 13827 | 20693418 | 0.067 | 1.98 (1.78,2.21) | <0.001 | | 1.56 (1.39,1.74) | | <0.001 |
| **Mortality,** *10-day risk period, N=109,024^c^ patients with 109,024 events and 1,174,775 observation periods* | | | | | | | | | |
| Fluoroquinolone Risk Period | 2621 | 1805210 | 0.145 | 1.00 |  | | 1.00 | |  |
| Fluoroquinolone vs. Amoxicillin^b^ | 500 | 583916 | 0.086 | 1.30 (1.18,1.44) | <0.001 | | 1.23 (1.12,1.36) | | <0.001 |
| Fluoroquinolone vs. Azithromycin | 246 | 348478 | 0.071 | 1.61 (1.41,1.84) | <0.001 | | 1.78 (1.55,2.03) | | <0.001 |
| Fluoroquinolone vs. Cefuroxime/Cephalexin | 216 | 314454 | 0.069 | 1.60 (1.39,1.84) | <0.001 | | 1.38 (1.20,1.59) | | <0.001 |
| Fluoroquinolone vs. Doxycycline | 232 | 295839 | 0.078 | 1.37 (1.19,1.57) | <0.001 | | 1.29 (1.13,1.48) | | <0.001 |
| Fluoroquinolone vs.  SMX-TMP | 289 | 344459 | 0.084 | 1.37 (1.21,1.55) | <0.001 | | 1.23 (1.09,1.39) | | <0.001 |
| Fluoroquinolone vs.  Other Antibiotics | 414 | 438195 | 0.094 | 1.20 (1.08,1.34) | 0.001 | | 1.06 (0.95,1.18) | | 0.30 |
| Fluoroquinolone vs.  Multiple Antibiotics | 629 | 394791 | 0.159 | 0.86 (0.79,0.94) | 0.001 | | 0.83 (0.76,0.91) | | <0.001 |
| Fluoroquinolone vs.  No Antibiotics | 103877 | 110983842 | 0.094 | 1.10 (1.05,1.14) | <0.001 | | 0.79 (0.76,0.83) | | <0.001 |

SCCSA=self-controlled case series analysis; IRR= incidence rate ratio; SMX-TMP=sulfamethoxazole-trimethoprim

^a^Adjusted for time-varying covariates of age, fiscal year, and respiratory, urinary, and skin and soft-tissue infections

^b^The numbers in the rows that follow “fluoroquinolone risk period” are for the comparator antibiotics (e.g., amoxicillin, azithromycin).

^c^N=56 patients were removed due to only one observation period
